# Fabrication mechanism of friction-induced selective etching on Si(100) surface

**DOI:** 10.1186/1556-276X-7-152

**Published:** 2012-02-23

**Authors:** Jian Guo, Chenfei Song, Xiaoying Li, Bingjun Yu, Hanshan Dong, Linmao Qian, Zhongrong Zhou

**Affiliations:** 1 Tribology Research Institute, National Traction Power Laboratory, Southwest Jiaotong University, Chengdu, Sichuan Province, 610031, People's Republic of China; 2 School of Metallurgy and Materials, University of Birmingham, Birmingham, B15 2TT, UK

**Keywords:** friction-induced selective etching, nanofabrication, silicon

## Abstract

As a maskless nanofabrication technique, friction-induced selective etching can easily produce nanopatterns on a Si(100) surface. Experimental results indicated that the height of the nanopatterns increased with the KOH etching time, while their width increased with the scratching load. It has also found that a contact pressure of 6.3 GPa is enough to fabricate a mask layer on the Si(100) surface. To understand the mechanism involved, the cross-sectional microstructure of a scratched area was examined, and the mask ability of the tip-disturbed silicon layer was studied. Transmission electron microscope observation and scanning Auger nanoprobe analysis suggested that the scratched area was covered by a thin superficial oxidation layer followed by a thick distorted (amorphous and deformed) layer in the subsurface. After the surface oxidation layer was removed by HF etching, the residual amorphous and deformed silicon layer on the scratched area can still serve as an etching mask in KOH solution. The results may help to develop a low-destructive, low-cost, and flexible nanofabrication technique suitable for machining of micro-mold and prototype fabrication in micro-systems.

## Introduction

Due to its excellent mechanical and physical properties, monocrystalline silicon has been widely used in micro/nanoelectromechanical systems [1,2]. The typical microscale and nanoscale fabrication technique of silicon devices is photolithography [3]. Although it has a strong merit in mass production, photolithography is not suitable for flexible machining of micro-mold and prototype fabrication in micro-systems. Moreover, as the scale-down of device dimensions continues, traditional fabrication methods have difficulty in meeting the resolution requirement [4,5]. Therefore, it remains very essential to explore new nanofabrication methods especially for nanoscale silicon devices.

Recently, scanning probe microscopy lithography (SPML) has attracted much attention because of its atomic resolution, simplicity, and low cost [5-10]. Among various SPML techniques, a maskless nanofabrication technique by a combination of nanoscratch and wet chemical etching was developed [8-10]. Based on the scratch and post-etching method, nanolines have been successfully fabricated on a silicon surface [10]. However, as a typical friction-induced selective etching technique, the intrinsic fabrication mechanism was still far from being understood.

Due to the mechanical interaction and tribochemical reaction during scratching, the scratched area of the silicon surface may contain a superficial oxidation layer and the tip-disturbed silicon layer in the subsurface [11]. In the previous studies [12-15], some researchers suggested that the superficial oxygen-rich layer acted as a mask during KOH etching. Little attention has been paid to the tip-disturbed silicon layer in the subsurface [16]. In fact, the oxygen-rich layer is very thin, and the concentration of oxygen decreases sharply with the depth [17]. Therefore, the mask ability of the thin oxygen-rich layer may be limited, and the thick tip-disturbed silicon layer (from tens to hundreds of nanometers) may also serve as an etching mask in KOH solution.

In the present study, the effect of load and etching time on the fabrication of a nanostructure on silicon by scratch and post-etching was investigated. A range of nanopatterns were produced on silicon surfaces. To understand the fabrication mechanism involved, the cross-sectional microstructure of the scratched area was observed, and the mask ability of the tip-disturbed layer was studied. The results may provide a way towards developing a low-destructive, low-cost, and flexible nanofabrication technique.

## Materials and methods

**Figure 1:**
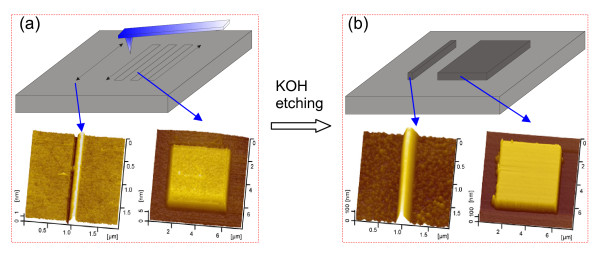
**Schematic drawing showing the fabrication process by friction-induced selective etching**. (**a**) Scratching a tip along the designed traces on the Si(100) surface, the two AFM images show the topography of the scratched area. (**b**) Produced nanostructures on Si(100) surface after KOH etching.

The p-type Si(100) wafers with a thickness of 0.5 mm were purchased from MEMC Electronic Materials, Inc. (St. Peters, MO, USA). Using an atomic force microscope (AFM; SPI3800N, Seiko, Tokyo, Japan), the root mean square roughness of the silicon wafers was measured to be 0.08 nm over a 500 × 500-nm area. The fabrication process was schematically shown in Figure 1. When the Si(100) surface was scratched by a tip along the designed traces, a groove or hillock may be generated on the scanned area depending on the applied contact pressure [18]. After KOH etching, different types of nanostructures can be produced on the target surface.

Some line scratches were produced on the Si(100) surface by a nanoscratch tester (CSM Instruments, Peseux, Switzerland) using a spherical diamond tip with a nominal radius of 5 μm at a sliding velocity of 5 μm/s. The scanning scratches were conducted using the AFM with a cubic diamond tip (Micro Star Technologies, Huntsville, TX, USA) at a scanning speed of 5 μm/s. The curvature radius of the tip was 400 nm, and the spring constant *k *of the cantilever was 194 N/m. Some nanopatterns were fabricated by an AFM (MFP-3D-SA, Asylum Research, Santa Barbara, CA, USA) with closed-loop scan function. After the scratch tests, the specimens were dipped in a mixture of 20 wt.% KOH and isopropyl alcohol (IPA) (volume ratio = 5:1) aqueous solution for post-etching [19]. All the AFM images of silicon specimens were scanned by silicon nitride tips (MLCT, Veeco Instruments Inc., Plainview, NY, USA) with *k *= 0.1 N/m. During scratching and etching, the experimental temperature was controlled at 20 ± 3°C and the relative humidity was varied between 50% and 55%. However, the small variation of experimental conditions, such as temperature and solution preparation, can influence the etching results, especially the etching height [20,21]. To acquire accurate results, each etching experiment was repeated at least three times.

**Figure 2:**
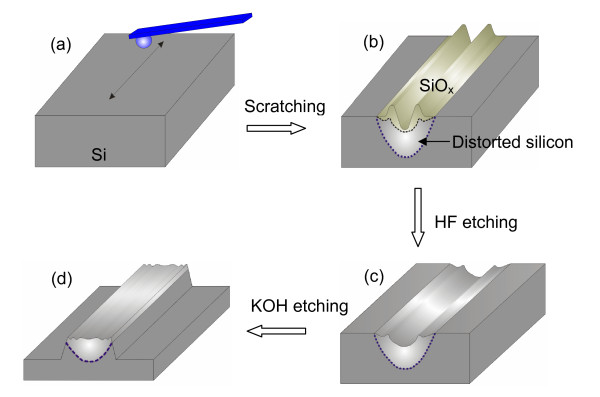
**Detection on the mask ability of the distorted silicon layer**. (**a**) Original Si(100) surface with native oxidation layer, (**b**) Si(100) surface after scratching, (**c**) Si(100) surface after etching in HF solution to remove surface oxidation layer. (**d**) Si(100) surface after etching in KOH solution.

To understand the fabrication mechanism of the friction-induced selective etching technique, the cross-sectional microstructure of the scratched area was characterized by a transmission electron microscope (TEM; FEG Philips Tecnai F20, FEI Company, Eindhoven, The Netherlands). The TEM sample was prepared by the focused ion beam technique. To protect the surface of the scratched area, a platinum layer with a thickness of about 200 nm was deposited on the sample surface by low-energy ion beam deposition. As shown in Figure 2, to study the mask ability of the tip-disturbed silicon layer, the line- and mesa-shaped nanostructures were fabricated on the Si(100) surface by scratching. After the removal of the oxidation layer on the surface of scratched area by etching in HF solution, the silicon samples were then etched in KOH solution to detect the mask behavior of the residual disturbed silicon layer. During these processes, the chemical composition of the mesas was detected by a scanning Auger nanoprobe (PHI 700, ULVAC-PHI, Inc., Chigasaki City, Kanagawa, Japan).

## Experimental results

### Load effect on the fabrication of nanolines on Si(100) surface

**Figure 3:**
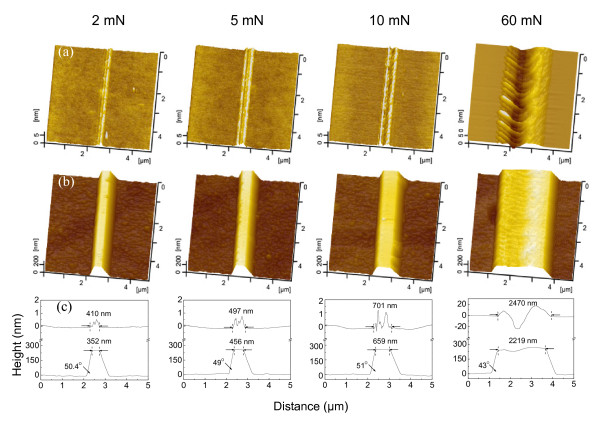
**Effect of load on the nanofabrication of Si(100) surface by scratching and post-etching**. (**a**) AFM images of the nanostructures on the Si(100) surface after scratching at various loads. (**b**) AFM images of the nanostructures on the Si(100) surface after post-etching in 20 wt.% KOH + IPA solution for 40 min. (**c**) Cross-sectional profiles of the nanostructures before (up) and after (down) post-etching.

In order to study the load effect on the fabrication of nanolines on silicon, nanoscratches were created on the Si(100) surface under various normal loads *F*_n_. As shown in Figure 3a, when the normal load was 2 mN, the corresponding Hertz contact pressure was estimated as 6.3 GPa. The scratch damage was very weak, and only a small protuberance was produced on the Si(100) surface. However, when the normal load was above 5 mN, the corresponding Hertz contact pressure was larger than 8.5 GPa. The scratch damage became severe, and the grooves were machined into the Si(100) surface. After etching in 20 wt.% KOH + IPA solution for 40 min, both protuberance and groove were evolved to nanolines with similar height, as shown in Figures 3b, c. The tilt angle of the etched sidewalls is about 50°, indicating that the incline sidewall is in the (111) plane [15].

It was noted that the width of the nanolines was strongly dependent on the width of the damage area on the Si(100) surface. With the increase in the normal load from 2 mN to 60 mN, the width of the fabricated nanolines on the Si(100) surface increases from 352 nm to 2,219 nm, which are well consistent with the width of the corresponding damage area before etching (from 410 nm to 2,470 nm). The results indicated that the width of the nanolines can be effectively controlled by the scratching load. Moreover, although the scratch under a normal load of 2 mN (the corresponding Hertz contact pressure is 6.3 GPa) induced a very weak damage on the Si(100) surface, it produced a nanoline with the similar height as that obtained by 60 mN after KOH etching. Therefore, a contact pressure of 6.3 GPa was enough to fabricate a mask layer on the Si(100) surface, and a low-destructive nanofabrication technique could then be developed based on this method.

### Etching time effect on the fabrication of nanolines on Si(100) surface

**Figure 4:**
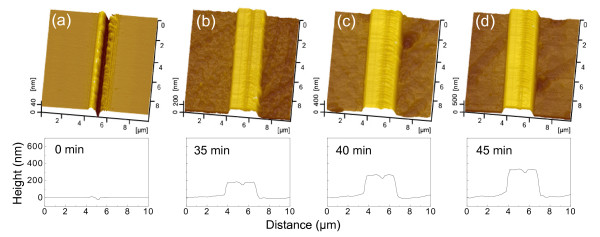
**Effect of etching time on the nanofabrication of Si(100) surface by scratching and post-etching**. The AFM image and cross-sectional profile of the nanostructure on the Si(100) surface (**a**) after scratching under applied normal load *F*_n _= 60 mN and (**b **to **d**) after post-etching in 20 wt.% KOH + IPA solution for 35 min, 40 min, and 45 min, respectively.

**Figure 5:**
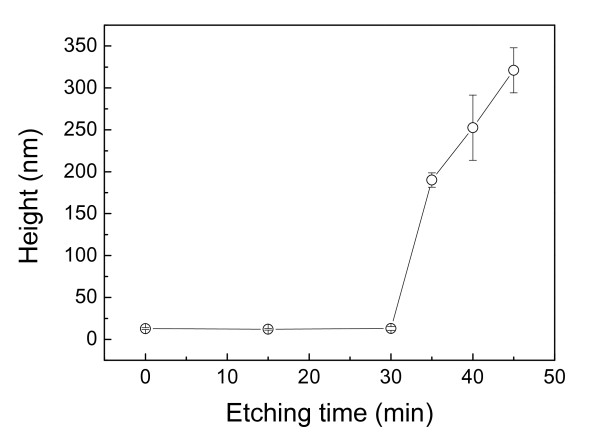
**Effects of etching time on height of the nanostructure on Si(100) surface by scratching and post-etching**. The scratching load is 60 mN, and the etching solution is 20 wt.% KOH + IPA solution. The error bars displayed the statistical results of the etching height.

Besides the scratching load, the etching time also shows a strong effect on the fabrication of nanolines on the Si(100) surface. After scratching on the Si(100) surface under a normal load of 60 mN, the specimens were etched in 20 wt.% KOH + IPA solution for various periods. Figure 4 shows the AFM images of the nanolines, and Figure 5 plots the variation of the height of these nanolines with etching time. Due to the mask effect of the native oxide layer, the height of the nanolines kept a very low value during the initial 30 min. After the native oxide layer was etched away, the height of the nanolines reveals a sharp increase with the increase of the etching time. Clearly, the scratched area on the Si(100) surface shows a strong mask ability to the etching of KOH solution. However, since the formation of the scratched area was the combined results of both mechanical interaction and tribochemical reaction, the scratched area of the Si(100) surface may contain a thin superficial oxidation layer and a thick tip-disturbed silicon layer in the subsurface [11,17]. Besides the superficial oxidation layer, the mask ability of the tip-disturbed silicon layer needed to be further investigated.

### Mask ability of structurally disturbed silicon

**Figure 6:**
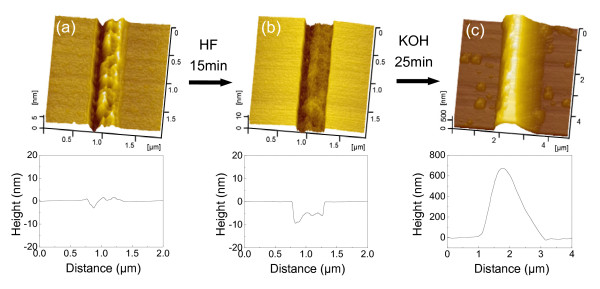
**The etching mask behavior of the distorted silicon layer on groove structure**. (**a**) Groove produced by one cycle of line scratch under *F*_n _= 5 mN on the Si(100) surface. (**b**) AFM image of the scratched area after etching in 10 wt.% HF solution for 15 min to remove surface oxidation layer. (**c**) AFM image of the scratched area after etching in 20 wt.% KOH + IPA solution for 25 min.

In order to detect the mask ability of structurally disturbed silicon, a groove structure was produced on the Si(100) surface after a line scratch under *F*_n _= 5 mN, as shown in Figure 6a. It was well known that an oxidation layer may be generated on the surface of the scratch area due to the tribochemical reaction. In the previous studies [12-15], such oxidation layer was believed to be the only mask structure on the scratch area. In fact, a thick distorted silicon layer including amorphous and deformed silicon may also be produced under the surface oxidation layer during the scratching process [11,17]. To study the mask ability of the distorted silicon layer in KOH solution, the surface oxidation layer of the Si(100) sample was removed by etching in HF solution for 15 min. As shown in Figures 6a, b, the depth of the groove increases from 3 nm to 8 nm after HF etching. Since the thickness of the oxidation layer on the scratch of the silicon surface was less than 5 nm, the oxidation layer formed on the groove during scratching should have been fully removed by HF etching [22]. When the sample was further etched in 20 wt.% KOH + IPA solution for 25 min, a hillock with the height of 710 nm was formed on the scratch area, as shown in Figure 6c. The results strongly suggested that besides the oxidation layer, the distorted silicon layer can also reveal excellent mask ability in KOH solution.

### Fabrication of nanopatterns on Si(100) surface

**Figure 7:**
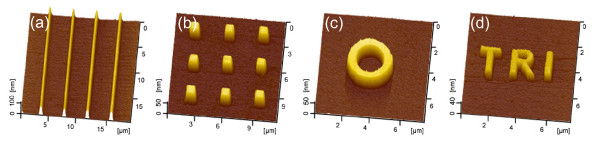
**Fabrication of nanopatterns on Si(100) surface by friction-induced selective etching technique**. (**a**) Protrusive array of nanolines by line scratch under *F*_n _= 2 mN and post-etching in 20 wt.% KOH + IPA solution for 2 min. (**b**) Protrusive array of nano-mesas produced by scanning scratch under *F*_n _= 15 μN and post-etching in 20 wt.% KOH + IPA solution for 2 min. (**c**) Nano-ring produced by scanning scratch under *F*_n _= 20 μN and post-etching in 20 wt.% KOH + IPA solution for 2 min. (**d**) Nanoword TRI produced by scanning scratch under *F*_n _= 20 μN and post-etching in 20 wt.% KOH + IPA solution for 1 min. The native oxidation layer on the Si(100) surface was removed by HF etching before scratching.

Based on the friction-induced selective etching method, a series of nanopatterns were fabricated on the Si(100) surface, as shown in Figure 7. In order to improve the fabrication efficiency, the native oxidation layer on the Si(100) surface was removed by HF etching before scratching. A protrusive array of nanolines with a height of about 100 nm (Figure 7a) was produced by line scratch under *F*_n _= 2 mN and post-etching in 20 wt.% KOH + IPA solution for 5 min. A protrusive array of nano-mesas with about 70 nm in height (Figure 7b) was fabricated by scanning scratch under *F*_n _= 15 μN and post-etching for 2 min. A nano-ring with a height of about 52 nm (Figure 7c) was produced by scanning scratch under *F*_n _= 20 μN and post-etching for 2 min, and a nanoword 'TRI' (short for 'Tribology Research Institute') with a height of about 31 nm (Figure 7d) was fabricated by scanning scratch under *F*_n _= 20 μN and post-etching for 1 min.

Therefore, through the control of scratch load, scan trace, and etching time, a nanopattern with the required shape, height, and width can be fabricated on the Si(100) surface with the present method. Compared with fabrication by the prevalent photolithography, this method introduces an optional fabrication approach with more flexibility and lower cost, which could provide new opportunities for the mold production in nanoimprint and prototype fabrication in micro-systems [23,24].

## Discussions

### TEM observation on the cross-sectional microstructure of scratched area

**Figure 8:**
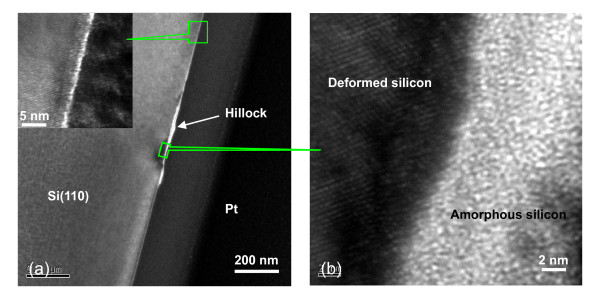
**XTEM detection on the cross-sectional microstructure of the scratched area of Si(100) surface**. (**a**) XTEM microstructures of the scratch produced by 300 cycles of line scratch under *F*_n _= 30 μN. The upper left inset picture shows the high-resolution microstructure of the original silicon surface. (**b**) High-resolution microstructure of the scratched area on the silicon surface.

To understand the fabrication mechanism of the friction-induced selective etching, the cross-sectional microstructure of the scratch on the Si(100) surface was studied by TEM, energy dispersive X-ray spectroscopy (EDX), and a scanning Auger nanoprobe. Figure 8a shows the cross-section TEM (XTEM) bright-field microstructures of the scratch sections. The inserted image in Figure 8a was taken from the single crystal area away from the scratched position. Figure 8b reveals that the scratched area is composed of a thin superficial layer and a relatively thick distorted (amorphous and deformed) case in the subsurface. Both EDX and scanning Auger nanoprobe analysis revealed that oxygen (from SiO_x_) can be only detected at the scratched surface areas, but cannot be found from the majority of the amorphous case and the severe deformed zone [25]. Therefore, the mechanical interaction through amorphization and matrix deformation have played a more important role than oxidation reaction during the scratching process on the Si(100) surface.

### Etching-resistant mechanism of structurally disturbed silicon layer

**Figure 9:**
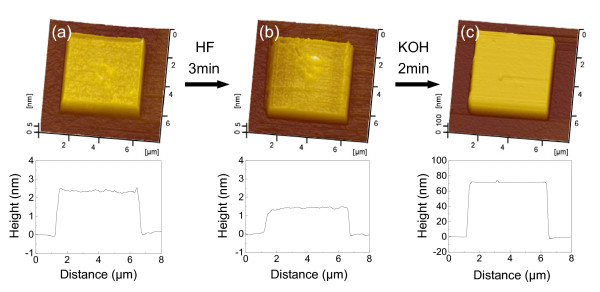
**The mask behavior of the structurally disturbed Si(100) surface in KOH solution**. (**a**) Mesa produced by 10 cycles of scanning scratch under *F*_n _= 50 μN on the Si(100) surface. (**b**) AFM image of the mesa area after etching in 10 wt.% HF solution for 3 min to remove surface oxidation layer. (**c**) AFM image of the mesa area after etching in 20 wt.% KOH + IPA solution for 2 min.

**Figure 10:**
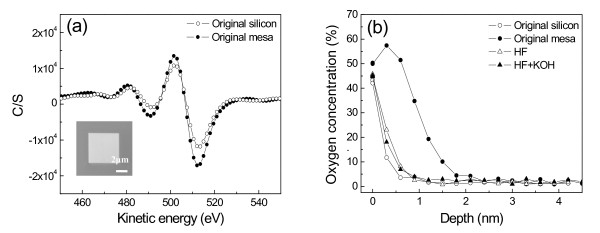
**Scanning Auger nanoprobe analysis on friction-induced mesa surface**. (**a**) The elemental composition of the friction-induced mesa and original silicon substrate. The lower left insert shows the SEM image of the tested mesa. (**b**) Oxygen atomic concentration distribution on four different surfaces: original silicon, original mesa, mesa etched in 10 wt.% HF solution for 3 min, and mesa etched in 10 wt.% HF solution for 3 min and then in 20 wt.% KOH + IPA solution for 2 min.

The above results suggested that the residual scratched structure can still resist against KOH etching after removing the superficial oxidation layer by HF etching. To further understand the etching-resistant mechanism of the structurally disturbed silicon layer, a scanning Auger nanoprobe was used to detect the variation of the chemical composition on the scratched area during the etching process. As shown in Figure 9, a mesa of 5 × 5 μm was fabricated on the Si(100) surface under *F*_n _= 50 μN and scratching cycles of 10 by AFM in a scanning scratch mode [25]. The height of the mesa was measured as 2.5 nm, as shown in Figure 9a. The scanning Auger nanoprobe analysis indicated that the intensity of oxygen on the mesa surface is higher than that on the original silicon surface (Figure 10a) and that the thickness of the oxidation layer is about 1.7 nm (Figure 10b). After the oxidation layer on the silicon surface was removed by etching in HF solution for 3 min, the height of the mesa decreased to 1.4 nm (Figure 9b). Due to the native oxidation of the silicon surface in air, the mesa showed a similar oxidation layer as that on the original silicon surface (Figure 10b). Finally, after etching in 20 wt.% KOH + IPA solution for 2 min, the height of the mesa sharply increased to 70 nm (Figure 9c). Therefore, since the native oxidation layer on the mesa is the same as that on the original silicon surface, the mask behavior of the mesa after etching in HF solution should be mainly attributed to the amorphous and deformed silicon induced by scratching.

It was well known that single-crystal silicon shows strong anisotropic etching behavior in alkaline solutions. Based on the electrochemical model proposed by Seidel et al. [21], the anisotropic etching behavior of silicon was mainly attributed to the differences on the density of the dangling bond as well as the structure and energy level of the backbond on the silicon surface. When the density of the dangling bond and the energy level of the backbond are high, the etching rate of the silicon surface in alkaline solutions is fast. Since {100} surfaces show the highest dangling bond density and a relatively high backbond energy level, the etching rate of {100} surfaces in alkaline solutions are the fastest. As a comparison, with one dangling bond and three backbonds per surface atom, the {111} surfaces show the lowest dangling bond density and the slowest etching rate in alkaline solutions. Finally, even with the similar dangling bond density as {111} surfaces, the {110} surfaces show a relatively higher backbond energy level and higher etching rate than {111} surfaces.

When the monocrystalline structure of silicon atoms on the Si(100) surface was transformed to amorphous or deformed silicon during the scratching process, the atomic arrangement on the surface became out of order. As a result, the average dangling bond density and backbond energy level of the silicon atoms on the distorted surface were lower than those of the original Si(100) surface [26]. Therefore, the amorphous and deformed silicon is relatively difficulty to be etched and can act as an etching mask of the Si(100) surface in KOH solution. The results may not only help us to understand the fabrication mechanism involved in the friction-induced selective etching technique, but also provide a way towards developing a low-destructive, low-cost, and flexible nanofabrication technique.

## Conclusions

In summary, the fabrication mechanism of friction-induced selective etching was investigated in this paper. The mask ability of structurally disturbed silicon in KOH etching has been validated. The main conclusions can be summarized as follows:

1. The height of the nanostructures was dominated by the etching time, while their width can be controlled by the scratching load. Since a contact pressure of 6.3 GPa was enough to fabricate a mask layer on the Si(100) surface, a low-destructive nanofabrication technique can then be developed based on this method.

2. TEM detection indicated that the cross-sectional microstructure of the scratch mainly contained a thin oxidation layer on the surface and a thick distorted (amorphous and deformed) layer in the subsurface.

3. Scanning Auger nanoprobe analysis indicated that the oxidation layer on the mesa could be removed by HF solution. The residual amorphous and deformed silicon layer can also exhibit strong mask ability in KOH etching.

## Abbreviations

AFM: atomic force microscope; EDX: energy dispersion X-ray spectroscopy; IPA: isopropyl alcohol; SPML: scanning probe microscopy lithography; TEM: transmission electron microscope; XTEM: cross-section transmission electron microscope.

## Competing interests

The authors declare that they have no competing interests.

## Authors' contributions

JG, CS, and BY finished the fabrication experiments and acquired the original data in this article. XL and HD have done the TEM observation and analysis. LQ and ZZ have made substantial contributions to the conception and design for this article. All authors read and approved the final manuscript.
